# MECHANISMS Study: Using Game Theory to Assess the Effects of Social Norms and Social Networks on Adolescent Smoking in Schools—Study Protocol

**DOI:** 10.3389/fpubh.2020.00377

**Published:** 2020-08-04

**Authors:** Ruth F. Hunter, Felipe Montes, Jennifer M. Murray, Sharon C. Sanchez-Franco, Shannon C. Montgomery, Joaquín Jaramillo, Christopher Tate, Rajnish Kumar, Laura Dunne, Abhijit Ramalingam, Erik O. Kimbrough, Erin Krupka, Huiyu Zhou, Laurence Moore, Linda Bauld, Blanca Llorente, Olga L. Sarmiento, Frank Kee

**Affiliations:** ^1^Centre for Public Health, Institute of Health Sciences, School of Medicine, Dentistry and Biomedical Sciences, Queen's University Belfast, Belfast, United Kingdom; ^2^Department of Industrial Engineering, Social and Health Complexity Center, Universidad de Los Andes, Bogotá, Colombia; ^3^Department of Public Health, School of Medicine, Universidad de Los Andes, Bogotá, Colombia; ^4^Queen's Management School, Queen's University Belfast, Belfast, United Kingdom; ^5^Centre for Evidence and Social Innovation, School of Social Sciences, Education and Social Work, Queen's University Belfast, Belfast, United Kingdom; ^6^Department of Economics, Appalachian State University, Boone, NC, United States; ^7^The George L. Argyros School of Business and Economics, Smith Institute for Political Economy and Philosophy, Chapman University, Orange, CA, United States; ^8^Behavioral and Experimental Economics Laboratory, School of Information, University of Michigan, Ann Abhor, MI, United States; ^9^School of Informatics, University of Leicester, Leicester, United Kingdom; ^10^MRC Social and Public Health Sciences Unit, University of Glasgow, Glasgow, United Kingdom; ^11^The Usher Institute and SPECTRUM Consortium, College of Medicine and Veterinary Medicine, University of Edinburgh, Edinburgh, United Kingdom; ^12^Fundación Anáas, Bogotá, Colombia

**Keywords:** smoking prevention, adolescents, mechanisms, social networks, social norms, game theory

## Abstract

This proof of concept study harnesses novel transdisciplinary insights to contrast two school-based smoking prevention interventions among adolescents in the UK and Colombia. We compare schools in these locations because smoking rates and norms are different, in order to better understand social norms based mechanisms of action related to smoking. We aim to: (1) improve the measurement of social norms for smoking behaviors in adolescents and reveal how they spread in schools; (2) to better characterize the mechanisms of action of smoking prevention interventions in schools, learning lessons for future intervention research. The *A Stop Smoking in Schools Trial* (ASSIST) intervention harnesses peer influence, while the Dead Cool intervention uses classroom pedagogy. Both interventions were originally developed in the UK but culturally adapted for a Colombian setting. In a before and after design, we will obtain psychosocial, friendship, and behavioral data (e.g., attitudes and intentions toward smoking and vaping) from ~300 students in three schools for each intervention in the UK and the same number in Colombia (i.e., ~1,200 participants in total). Pre-intervention, participants take part in a Rule Following task, and in Coordination Games that allow us to assess their judgments about the social appropriateness of a range of smoking-related and unrelated behaviors, and elicit individual sensitivity to social norms. After the interventions, these behavioral economic experiments are repeated, so we can assess how social norms related to smoking have changed, how sensitivity to classroom and school year group norms have changed and how individual changes are related to changes among friends. This Game Theoretic approach allows us to estimate proxies for norms and norm sensitivity parameters and to test for the influence of individual student attributes and their social networks within a Markov Chain Monte Carlo modeling framework. We identify hypothesized mechanisms by triangulating results with qualitative data from participants. The MECHANISMS study is innovative in the interplay of Game Theory and longitudinal social network analytical approaches, and in its transdisciplinary research approach. This study will help us to better understand the mechanisms of smoking prevention interventions in high and middle income settings.

## Introduction

### Adolescent Smoking Behavior

Globally, tobacco smoking constitutes the single most important preventable risk factor for chronic disease in high, middle and low income countries. While rates of smoking have declined in high income countries, they continue to rise or remain high in some low and middle income countries (LMICs) (1). The tobacco industry has recently focused more attention on LMICs, as its markets are eroded elsewhere. Smokers usually start as adolescents when the influence of social norms on behavior is most apparent. In schools in Bogotá, the current prevalence is the highest in the country, at 13.1% (2). Comparing the UK to Colombia, tobacco use prevalence among boys and girls aged 13–15 years old in Colombia is up to 6% points higher than in the UK (i.e., in Colombia 12% in boys and 9% in girls; in UK, 6% in boys and 8% in girls). The channels of influence on smoking behaviors can be direct and indirect, but peers consistently have a strong impact on behavior (3). As such, tobacco control interventions should target the age groups most susceptible to becoming lifelong smokers using the most effective channels.

Schools provide one setting for such interventions, particularly those aimed at shaping peer norms and interactions. In a recent systematic review of school-based smoking prevention interventions, only those with social competence and or social influence components were effective (4). However, most of the studies in that review were from high income settings. In another review of interventions evaluated in randomized controlled trials (RCTs) in LMIC settings, only three school based intervention trials invoked theories of social competence or social influence, and the authors concluded that any adaptation of evidence from high income settings must rest on a careful analysis of contextual factors to achieve similar results elsewhere (5). Even though *peer influence* is often a component of effective smoking prevention interventions (such as the *A Stop Smoking in Schools Trial* (ASSIST) intervention) (6), when Steglich et al. *modeled* peer influence in a subset of ASSIST schools (*n* = 3) using actor-based social network models, they acknowledged the need for future research to explore *how* peer influence varied across individuals and across schools (7).

### Social Networks and Social Norms

In order to develop better public health interventions, it is important to understand the mechanisms by which they exert their effects. Interventions that act on groups of people or whole populations may be more effective at reducing health inequalities than those that target individuals (8). Changing social norms is one way of affecting behavior in groups of people, but these social norms often depend on connections and shared “understandings” between members of the population. The connections between people are what characterize their shared “social network,” and this may affect the way that social norms spread among people. Public health scientists know surprisingly little about how best to measure and evaluate the spread of social norms and their effects on behavior.

The past decade has seen improved methodological rigor and a deeper understanding of both the theories and techniques of behavior change (9). There are clearly certain categories of population-level interventions whose effects are realized, at least in part, through changing social norms. Social norms themselves may depend on context or be conditioned and shaped by social networks. Thus, they span different levels of what is more broadly referred to as the “socio-ecological model” of behavior change (10). There has been a parallel and growing interest in understanding the effects of social networks on health related behavior (11–13).

Two competing theories have been offered about how health-related attitudes and behavior evolve and are transmitted across social networks, namely: (i) *selection* (homophily)—the tendency for people to establish relations with those they perceive to be most like (similar to) themselves; and (ii) by *influence*, whereby one adopts the behaviors that are seen as normative within the peer environment. Most observational studies show that both processes operate to varying degrees. Valente and Pitts (14) have recently articulated social networks' influence on behavior in terms of “the pressure individuals feel to conform to behavior when others in equivalent positions do so” and offers two putative mechanisms: (i) one via threshold effects whereby, for example, “low threshold” individuals adopt a new behavior before many of their peers have done so; (ii) and a second that weights “exposure” (and influence) according to structural characteristics of the network. While social networks are increasingly being exploited in the design and implementation of public health interventions (8, 15), a growing number of studies are now also testing interventions based on changing social norms related to health behaviors (16).

Within public health science, there have been few empirical studies exploring the *mechanisms* underlying the influence of social norms on health related attitudes and behavior. *Game Theory* is a branch of economics that has developed a canon of well-defined mathematical models for describing and understanding cooperation and competition amongst individuals and groups. However, while Game Theorists have increasingly adopted experimental approaches to test their more sophisticated models of social norms effects (17), the resulting insights have not crossed the disciplinary divide into the public health sciences. Previous attempts at identifying individual sensitivity to the effects of social norms have relied on actor-based *models* and have not actually attempted to empirically measure the effects directly. An experimental design rooted in Game Theory offers new ways to do this, and provides the opportunity to explore the behavioral economic mechanisms underlying the influence of social norms on health related attitudes and behavior.

### The Role of Game Theory Experiments in Studying Social Norms and Peer Influence

Kimbrough et al. (18) show that one can use simple rule following games to identify individual sensitivity to social norms. This norm sensitivity is a good explanation for cooperation, reciprocity and prosocial behavior in *Trust, Public Goods, Dictator*, and *Ultimatum* Games. Krupka et al. (20) use norm elicitation experiments to show that informal agreements affect behavior through a direct effect on the social norm and through an indirect effect by which the social norm appears to influence beliefs. These findings are significant because they identify a channel for behavior change that operates through a change in norms and beliefs at the individual level. This channel is a social channel: the norm is contingent on the peers who establish, transmit, and enforce the norm. However, previous work has not yet tested this channel where it can be most effective—nested within the social network of the individual. This study will test this channel within a social network. We will identify the impact of an intervention designed to change norms and beliefs. We will focus on smoking prevention in adolescents because it is an important public health concern and a critical population to reach.

The key objectives of this study are to:

1. develop and test new measures of social norms around smoking behaviors in adolescents using Game Theory approaches;
2. better understand the diffusion of social norms in school settings in the UK, and in Colombia;
3. better characterize the potential mechanisms of action of smoking prevention interventions in schools;
4. learn lessons for the design and evaluation of behavior change interventions that invoke mechanisms that change social norms.

Our main research questions include:

1. How are individual psychosocial and cognitive traits at baseline related to individual sensitivity to social norms? (**H**_**0**_^**1**^: that they are independent);
2. How does individual sensitivity to social norms cluster among friendship cliques and across school year groups? (**H**_**0**_^**2**^: that the individual social norms sensitivities among friendship cliques are un-correlated);
3. How are average social norms, measured at the classroom and year group level, affected by social network structures? (**H**_**0**_^**3**^: that social norms and social network structures are independent);
4. After each intervention: how are changes in attitudes, intentions and behaviors toward smoking related to social norms sensitivity at the individual level, and to average social norms at the class and year group level? (**H**_**0**_^**4**^: that the changes in smoking related attitudes, intentions, and behaviors are independent of norm-sensitivity at the individual level and the same in schools which offered each intervention);
5. After each intervention: have smoking-related social norms changed and how are these changes correlated among friendship cliques? (**H**_**0**_^**5**^: that any changes in social norms are independent among members of the same friendship clique; we assume that there will be little change in measures of homophily across one semester).

## Methods and Analysis

The protocol follows the Standard Protocol Items: Recommendations for Interventional Trials (SPIRIT) guidelines (21).

### Study Design

Our study is a quasi-experiment with a before and after design. The interventions have both been evaluated in separate cluster RCTs (6, 22). However, our proposed studies are not conceived to be “head-to-head” comparisons of their effectiveness. The studies, rather, are *mechanism focused* and the two school-based interventions offer contrasting conditions: one leverages peer influence and the other uses a more straightforward classroom approach to deliver information. While ultimately a classroom and school based norm around smoking may change for each intervention, the change mechanism may be different.

Using a whole school year approach, our study design involves an investigation of the evolution of social networks and norms around smoking before and after a smoking prevention intervention. First, we conducted a cultural adaptation and a pilot study in three schools (one school in Northern Ireland and two schools in Colombia) with 312 pupils during 2018–2019. In 2019, in each country (Northern Ireland and Colombia) we studied ~600 pupils (aged 12–13 years/Year nine pupils in Northern Ireland and aged 12–15 years/Year seven in Colombia) in six secondary (i.e., post-primary) level schools (three receiving each intervention) exploring the contrasts between schools receiving different interventions and between countries (where norms are different).

### Interventions

The interventions were culturally adapted for use in Colombia to fit the needs of the local context while keeping their fidelity and avoiding “cultural hegemony” bias. The cultural adaptation process included four stages (information gathering, preliminary adaptation design, preliminary adaptation test, and adaptation refinement) (23). Furthermore, all the materials for data collection were translated and back translated by bilingual speakers/translators.

#### ASSIST (A Stop Smoking in Schools Trial) Intervention

**Figure 1:**
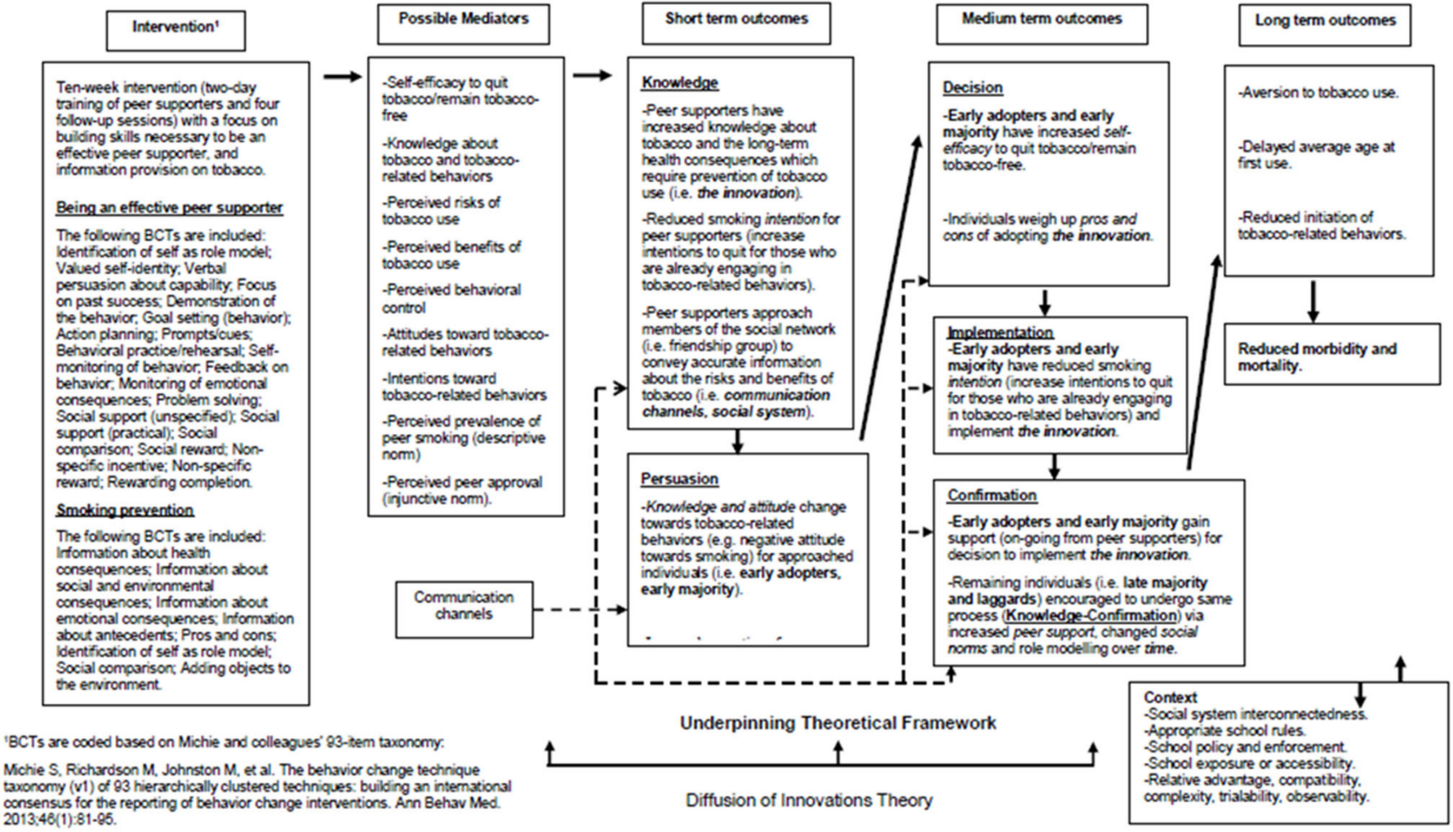
Proposed logic model for the ASSIST intervention.

The ASSIST intervention is designed to train influential pupils to use informal contacts with peers in their school year group to encourage them not to smoke (24, 25). The effectiveness of the ASSIST intervention for smoking prevention has previously been established in a cluster RCT (6). Figure 1 shows the proposed logic model depicting assumed pathways of change for participants receiving the ASSIST intervention. Underpinned by Diffusion of Innovations Theory (26), its core elements include the identification and recruitment of peer supporters (through a process of nominating pupils), who are then trained to diffuse prevention messages through informal conversations with their classmates.

According to Rogers, diffusion is “the process in which an innovation is communicated through certain channels over time among the members of a social system” (26). Therefore, the four key components in the diffusion of an innovation is the innovation (e.g., smoking prevention message), communication channels (e.g., one-to-one conversations by peer supporters in their friendship groups), time (e.g., the period of time during which the message is being spread), and the social system (e.g., the school year group). The five stages of the innovation-decision process, leading to adoption (i.e., “full use of an innovation as the best course of action available”) or rejection (i.e., a decision “not to adopt an innovation”) of the smoking prevention message for each individual in the school year group are: (1) Knowledge; (2) Persuasion; (3) Decision; (4) Implementation; and (5) Confirmation. Uncertainty in adoption is reduced when individuals are well-informed about the pros and cons of adopting the message. Therefore, the communication channels (i.e., one-to-one conversations between peer supporters and their friends) are vital to progress the innovation-decision process, from the “Knowledge” stage to the “Confirmation” stage, for all members of the school year group. In the short term, it is hypothesized that the intervention will directly lead to increased knowledge regarding tobacco and its long-term health consequences for peer supporters. Therefore, peer supporters will have reduced intentions to engage in tobacco-related behaviors. During the “Knowledge” stage, peer supporters approach members of their social network (i.e., friendship groups) to convey accurate information about the risks and benefits (i.e., pros and cons) of tobacco use. During the “Persuasion” stage, early adopters and early majority (i.e., well-connected individuals who are first to receive the message), undergo a knowledge and attitude change toward tobacco-related behaviors (e.g., negative attitude toward smoking) and have increased perceptions of peer support. In the medium term, self-efficacy to quit tobacco, or remain tobacco-free, should be increased by increases in social support (i.e., vicarious experience) (27). During the “Decision” stage, early adopters, and early majority weigh up the pros and cons of adopting the message (28). Increased knowledge, attitude changes, and perceptions of peer support influence the early adopters and early majority toward implementing the innovation and reducing their smoking intentions during the “Implementation” stage (29, 30). During the “Confirmation” stage, they gain support (on-going from peer supporters) for the decision to implement the innovation. Furthermore, in the medium term, the remaining individuals in the social system (i.e., late majority and laggards) who are less well-connected than the early adopters and early majority are encouraged to undergo the same process (i.e., to progress from the “Knowledge” to the “Confirmation” stage) due to increased perceptions of peer support, changed social norms, and role modeling (27, 31). In the longer-term, the intervention is expected to lead to reduced rates of initiation of tobacco-related behaviors, delayed average age at first tobacco-use, reduced morbidity and mortality, and improved health and mental well-being (32–35).

According to the Diffusion of Innovations Theory, contextual factors influencing the rate of adoption include: relative advantage (i.e., the degree to which an innovation is perceived as being better than the preceding idea); compatibility (i.e., the degree to which an innovation is perceived as being consistent with existing values, experiences and needs of adopters); complexity (i.e., the degree to which the innovation is perceived as being difficult to understand and use); trialability (i.e., the degree to which an innovation may be experimented with on a limited basis); and observability (i.e., the degree to which the results of the innovation are observable to others via role modeling for example) (27, 36).

All pupils in participating schools are asked to complete a peer questionnaire to nominate up to five pupils they view as influential in different respects (i.e., whom they respect, who they think are good leaders in sports or other group activities, whom they look up to) in their year at their school. The top 18% nominated pupils in each year group are invited to train and take on the role of “peer supporter.” The intervention provides peer supporters with the knowledge and skills necessary to spread information about the harms of tobacco-use amongst the remaining members of the school year group (i.e., peer education and diffusion).

Broadly, the 2-days peer supporter training course aims to increase knowledge about the health, economic, social, and environmental risks of smoking; emphasize the benefits of remaining smoke-free; and encourage the development of skills to enable peer supporters to promote non-smoking among their peers. The training program consists of three broad categories of information giving, communication skills, and personal development. After the peer supporter training, peer supporters intervene informally in everyday situations over a 10-weeks period to encourage their peers not to smoke, and to keep a diary record of these informal conversations. Trainers make four follow-up visits to each school over this 10-weeks period to provide further training to the peer supporters and to monitor their progress. The detailed protocol for the ASSIST intervention was developed by Evidence to Impact (http://evidencetoimpact.com/), a social enterprise. The developers of ASSIST visited Bogotá in order to assess fidelity of the intervention.

#### Dead Cool Intervention

Dead Cool is a smoking prevention intervention for pupils aged 12–13 years old designed by Cancer Focus Northern Ireland (a local cancer charity) (22, 37). The intervention is designed to be delivered by teachers and consists of eight lesson plans and an accompanying DVD of short video clips to supplement each lesson. The intervention aims to reduce the number of young people who start smoking and to examine the influences on smoking behavior from friends, parents, other family members, and the media. Teachers from the school deliver the intervention in their own classes over an 8-weeks period. The lessons last for ~20–30 min. Teachers have a Teachers' Resource Pack and pupils have a Pupil Workbook that contains exercises for each lesson. Before the start of the intervention, pupils have an introductory session to the intervention and teachers receive professional development that outlines the focus and epistemology behind the product design.

**Figure 2:**
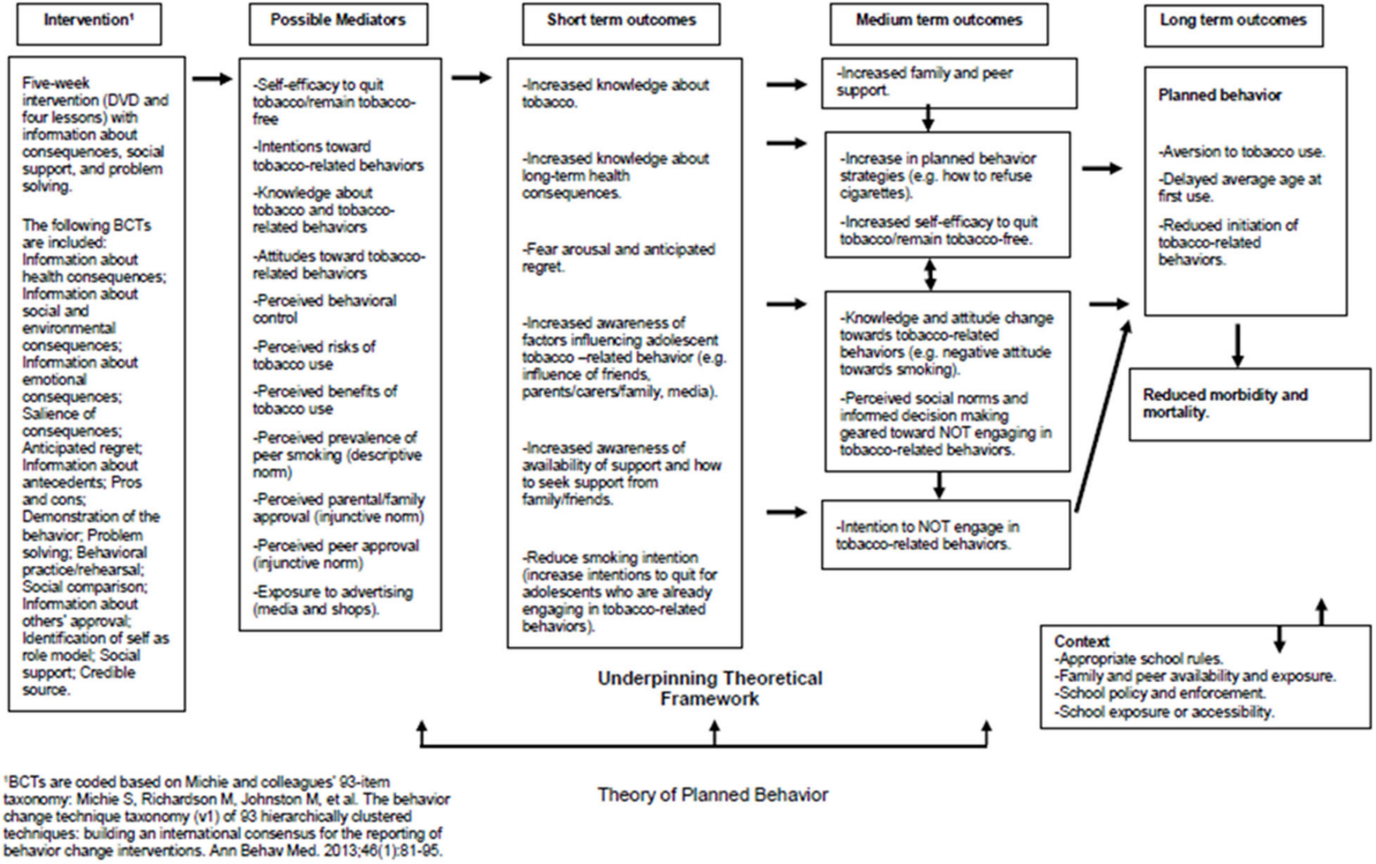
Proposed logic model for Dead Cool intervention.

Figure 2 shows the proposed logic model depicting assumed pathways of change for participants in the Dead Cool intervention. The intervention is motivated by more conventional classroom pedagogy and the Theory of Planned Behavior (29, 30). The theory has elsewhere been tested and evidence suggests that it delays or prevents smoking initiation (38). The intervention includes a range of behavior change techniques (BCTs) including provision of information about consequences, information about behavioral antecedents (e.g., influence from friends, family, and the media), problem solving, and social support. In the short-term it is hypothesized that the intervention's information provision components should lead directly to increased knowledge about tobacco and the long-term health consequences, with emphasis on the salience of outcomes leading to fear arousal and anticipated regret (39, 40). Provision of normative information regarding the prevalence of smoking and tobacco related behaviors for the participants' age group should reduce “misperceptions” and act to align perceived social (descriptive) norms with actual norms (41–43). The intervention should lead to increased awareness of the sources of support available and of how to seek support from family and friends. Self-efficacy to quit tobacco, or remain tobacco-free, should be increased by increases in social support (i.e., vicarious experience) (27). In the medium term, increases in perceived social support from family and peers, changing attitudes toward tobacco-related behaviors (e.g., negative attitude toward smoking) and changes in perceived social norms should lead participants to form intentions not to smoke or engage in tobacco-related behaviors (29, 30). In the longer-term, the intervention is expected to lead to reduced rates of initiation of tobacco-related behaviors, delayed average age at first tobacco-use, reduced morbidity, and mortality, and improved health and mental well-being (32–35).

In a pilot cRCT (37) in 18 schools (20 classes, 399 pupils, Year 9), Dead Cool was effective at reducing smoking susceptibility and carbon monoxide (CO) validated smoking behavior; (standardized effect size 0.38). However, there was unexplained school level heterogeneity in the effects (22). It has been proposed that social norms and peer influences may moderate some of the effects of such interventions (44). However, the Dead Cool intervention does not specifically train peer supporters to deliver prevention messages in the way that the ASSIST intervention does. The developers of Dead Cool visited Bogotá in order to assess fidelity of the intervention.

This team conducted an independent observational study of schools in Northern Ireland (*n* = 17) (Belfast Youth Development Survey). We employed the same actor-based hidden Markov models as Steglich et al. (7) and were able to demonstrate distinct school level heterogeneity in the effects of social networks on smoking behavior (45). Thus, both the ASSIST and Dead Cool studies suggest that local contextual factors might moderate or mediate some of their effects.

#### Intervention Delivery and Fidelity Checking

The ASSIST intervention was delivered by health promotion trainers from the local public health agency in respective countries who had participated in the ASSIST “Train the Trainer” sessions which enabled them to deliver the intervention in a standardized manner (6, 25). This is a 3-days session delivered by Evidence to Impact. The Dead Cool intervention was delivered by teachers in participating schools with an introductory session delivered by a Cancer Focus Northern Ireland employee (22, 37). In Bogotá, Dead Cool was delivered by trainers from the Universidad de Los Andes. Prior to the intervention, teachers in Northern Ireland received ~120 min of professional development. The teachers from Bogotá received a 2-days workshop (16 h) on professional development.

Up to three of the training and intervention sessions in Bogotá were video-taped to check intervention fidelity. The audio of the video tapes was transcribed and translated to English to enable project partners at Cancer Focus Northern Ireland and Evidence to Impact (who developed the interventions) to check for intervention fidelity. The translated transcripts were viewed alongside the video tape. Intervention fidelity and delivery were assessed by collecting data from trainers and teachers implementing the Peer Supporter Training and Follow-Up sessions. Fidelity checks include: pupil attendance, activity coverage, activity completed, lesson cover, lessons completed, content covered, and use of extension activities.

### Recruitment

#### Recruitment of Schools

Six post-primary schools were recruited in Northern Ireland and six in Bogotá during 2019. In Northern Ireland, schools were selected to ensure a mix of State and Catholic maintained schools, with some serving urban and rural catchments, and maximum variation sampling to ensure schools with high and low proportions of pupils eligible for free school meals. In Bogotá, six public schools from low to middle socio-economic status with maximum variation by geographic location were selected.

In Northern Ireland, relevant schools were identified from the database of schools which have previously been involved in research with the Center for Public Health and School of Education (currently over 200 schools in Northern Ireland). Schools were selected based on the sampling framework highlighted above. Schools identified as eligible were sent a letter (addressed to the school Principal or relevant senior member of staff) outlining the purpose of the study and inviting them to take part. A follow-up phone call by a member of the research team, ~1 week after delivery of the invitation letter, provided further details and an opportunity to answer any questions. For those schools agreeing to take part, a study pack was delivered providing further details about the study and role of the school.

In Bogotá, the sampling of schools followed these steps. First, a list of 40 private and public schools were prioritized based on health risks by the Education and Health Departments of Bogotá. Second, from this list, 13 schools were invited to participate according to the following inclusion criteria: (1) schools in an urban area; (2) including boys and girls; (3) having an enrollment of between 90 and 150 students in 7th year. Third, only six schools accepted the invitation and were selected for the final sample. These schools were assigned randomly to each of the interventions. Three schools were assigned to the ASSIST (*Entre Parceros*) intervention, and the other three were assigned to the Dead Cool (*Bacan*í*simo*) intervention. Schools identified as eligible were visited multiple times and researchers presented the outline of the study to the school Principal and to relevant members of staff, and invited them to take part. For those schools agreeing to take part, a study pack was delivered providing further details about the study and role of the school.

#### Recruitment of Participants

For each school recruited, we aimed to recruit all classes in a single year group (~*N* = 80–100 on average per school; study *N* = ~600 for each country). In each school, we targeted Year 9 pupils in Northern Ireland and Year 7 in Bogotá, who were 12–13 years old. All pupils in the school year were invited to participate. Prior to the conduct of the Game Theory Experiments, each school was given Teacher Information Sheets, Pupil Information Sheets, Parent/Guardian Information Sheets, Pupil Consent Forms, and Parent/Guardian Opt-Out Forms (providing information about the study). All pupils were required to complete Consent Forms indicating whether they agree or decline to participate. In Northern Ireland, Parents/Guardians who did not wish their child to take part were asked to return completed Opt-Out Forms.

### Sample Size

As this is a proof of concept study, we have provided an exemplar power calculation to assess how the social network clustering of smoking-related social norms might change after a prevention intervention (${\text{H}}_{0}^{5}$). This draws on the work of Krupka who studied ~200 Michigan university freshmen before and after a single semester. Krupka did not have data on smoking *per se* among the freshmen, but did have their elicited time and risk preferences. These measures of preferences are more fundamental behavioral economic traits that are correlated with smoking behaviors (46). Using the basic Clauset et al. community detection algorithm (47), with a sample size of ~200, and with data from two waves, Krupka found that a one standard deviation (*SD*) increase in risk preferences of an individual's friends or social network is associated with an increase of 1/8 to 1/10th of a SD (of the same variable) for the individual. Assuming that clustering of risk preferences is a reasonable proxy for clustering of social norms related to smoking that we will elicit, we estimate that a sample size of 300 would give over 80% power to detect, as statistically significant at the 5% level, a slope of 0.16. In other words, an increase of 0.16 *SD*s in individual norms sensitivity, per *SD* increase in norms sensitivity of those in the individual's social network.

### Outcomes and Data Collection Methods

Pupils who consented to participate were asked to complete a baseline assessment. This involved participating in a series of game theory experiments and completing a self-report questionnaire.

#### Game Theory Experiments

In order to identify social norms and their role in supporting smoking prevention interventions in schools, we triangulate the findings from a number of different game theory experiments, conducted before and after the smoking prevention interventions. In all of these incentivized experiments, monetary amounts were presented in cash in Northern Ireland and a gift card in Bogotá. Game Theory Experiments and questionnaires were delivered via Qualtrics (www.Qualtrics.com) and collected using tablet computers in each school.

##### Identifying general norms sensitivity

We employed an individual decision task (a variant of the Rule-Following, or RF Task) (18, 19) that measures participants' preferences for following established rules and social norms, in a context entirely removed from peer interaction. Specifically, we tell participants to follow an arbitrary rule when doing so provides them with no monetary benefits and instead imposes explicit monetary costs proportional to the degree of rule following.

Participants sequentially allocate 50 balls across two buckets (one blue and one yellow) and they are instructed that “The rule is to put the balls in the blue bucket.” Participants can choose freely whether to follow the rule and there are no consequences for breaking the rule. Participants know that they receive tokens to the value of 5 pence in Northern Ireland and 2 pence in Colombia for each ball they place in the blue bucket and 10 pence in Northern Ireland and 4 pence in Colombia for each ball they place in the yellow bucket. Therefore, a participant could earn £5.00 in Northern Ireland and £2.30 in Colombia if he/she ignores the stated “rule” and placed all 50 balls in the yellow bucket. On the other hand, if he/she followed the rule completely he/she would earn £2.50 in Northern Ireland and £ 1.14 in Colombia. The value of the tokens in Colombia was established according to the market and to prevent coercion. Participants were informed that they had 7 min to allocate the 50 balls between the two buckets and that any balls which are not allocated by the end of the 5 min are worth nothing. No other information, apart from the payment scheme and a general description of the procedure, was provided in the instructions.

As previously validated (18), the extent of rule-following in the RF task provides a measure of individual norm-following proclivity and this norm sensitivity measure is a good explanation for cooperation, reciprocity and prosocial behavior across decision contexts (18). In this task, an individual gets the largest payoff by breaking the rule and the smallest payoff by following the rule completely. Thus, the more a participant cares intrinsically about rule-following the more willing he/she will be to incur costs of doing so (18). The choices that participants make allow us to test whether those who reveal a stronger preference for following norms will be more likely in other contexts to be influenced by social norms (as was demonstrated in the original study) (18).

##### Measuring injunctive and descriptive norms

Injunctive norms reflect shared beliefs about what actions people *ought to* take; descriptive norms reflect shared beliefs about the prevalence of the norm (rather than beliefs about the behaviors that ought to be influenced by the norm). To measure injunctive and descriptive norms, we followed a protocol developed by Krupka and Weber (17). In a series of Coordination games, participants rated the social appropriateness of various actions that others might take and, in addition, they estimated the frequency with which people actually engage in certain behaviors. These games provide respondents with incentives to *match* their ratings/estimates to the responses of other participants in their year group in the session rather than to provide their personal opinions. In summary, we are measuring first-order beliefs about the prevalence of a norm against smoking/vaping and second-order beliefs about the social appropriateness of various smoking/vaping related behaviors.

The protocol measures injunctive norms because it creates an incentive to anticipate the extent to which others will rate an action as socially appropriate or inappropriate, and to respond accordingly. That is, if there is a social norm that some actions are more or less socially appropriate, respondents attempting to match others' appropriateness ratings are likely to rely on this shared perception to help them do so. Thus, the protocol elicits collective perceptions of appropriateness—our empirical measure of an injunctive social norm. This protocol was adapted to measure injunctive norms of prosocial behavior, injunctive norms about smoking behavior, and descriptive norms about smoking behavior. To encourage participants to *match* their ratings/estimates to the responses of other participants in their year group, they were paid £10.00 in Northern Ireland and £3.43 in Colombia for one “part” in the experiment only if their response was the same as the most common response given by other participants for a randomly selected question in that part of the experiment. For each “part” of the experiment, participants were told that at the end of the intervention, a coin would be flipped to determine whether they received their earnings from the baseline experiment or the experiment conducted after the intervention.

##### Defining and identifying social norms unrelated to smoking

We first elicited pro-sociality norms unrelated to smoking behavior (as one type of negative control). We used a vignette describing a hypothetical Dictator game scenario as per Krupka and Weber (17) and Kimbrough et al. (18). The Dictator game is commonly used as a measure of social preferences, in particular, altruism (48). Such norms are unlikely to be affected by interventions targeted at altering smoking behavior. Hence, we measured norms of altruism before and after the intervention, as a “negative control.” We do not expect altruism norms to change after the smoking prevention interventions. In our norm elicitation experiment participants read a vignette that describes the choices an “Individual A” would be faced with, within the Dictator Game. “Individual A and Individual B from the class are randomly paired with each other. Individual A received £10.00 in Northern Ireland and £2.30 in Colombia. Individual A will then have the opportunity to give any amount of his or her money to Individual B. For instance, Individual A may decide to give £0.00 to Individual B and keep £10.00 for him or herself. Or Individual A may decide to give £10.00 to Individual B and keep £0.00 for him or herself. Individual A may also choose to give any other amount between £0.00 and £10.00 to Individual B. This choice will determine how much money each will receive, privately and in cash, at the end of the experiment.” We used a Coordination Game, as described above, to elicit the social appropriateness of each action available to “Individual A,” measuring injunctive norms of pro-sociality before and after the intervention.

##### Measuring injunctive social norms related to smoking

To measure injunctive norms about smoking behavior (i.e., beliefs about the appropriateness of smoking and vaping related actions), we used another Coordination Game. The actions evaluated in this game include: (i) a parent smoking in their own home in front of their children who are under the age of five; (ii) an adult smoking in a car with children under the age of 16 in the car; (iii) someone selling cigarettes to a teenager who looks younger than 16 without requesting proof of age; (iv) a lead actor seen smoking in the opening scene of a recent superhero movie; (v) an older school pupil smoking outside school (e.g., at a bus-stop); (vi) a school pupil using an e-cigarette while walking to school; (vii) a pupil sharing a photo of himself/herself using an e-cigarette on social media (e.g., Facebook, Instagram); (viii) a school pupil using chewing tobacco. We adapted the Krupka and Weber (17) protocol to elicit beliefs about the injunctive norm by asking participants to coordinate with their year group peers in estimating the social appropriateness of various smoking related behaviors on a six point Likert scale that ranges over “extremely socially inappropriate” to “extremely socially appropriate” (i.e., as above their pay-off in the choice task is determined by whether their response matches the most common response given by the year group). By undertaking this Coordination game both pre- and post-intervention, we can identify whether or not some of the target norms (e.g., attitudes related to peer smoking) have changed, while acknowledging that injunctive norms for other behaviors [defining and identifying social norms unrelated to smoking] are not anticipated to be affected by the prevention programs (negative controls). This allows us to create one type of metric with which to understand the effects of the interventions on classroom social norms.

##### Elicitation of expected behavior by others (descriptive norms) related to smoking

To measure a respondent's beliefs about what his/her peers expect of one another regarding smoking and vaping behavior, we adapted the Krupka and Weber protocol (17) to elicit beliefs about expectations that others have for their peers by asking participants to coordinate on a six point Likert scale that ranges from whether: “most of my peers would be accepting of -[behavior]-;““1 = None of my peers; 2 = Only a few of my peers; 3 = Some of my peers; 4 = A lot of my peers; 5 = Most of my peers; 6 = All of my peers.” The personal behaviors being judged here are (i) smoking; and (ii) vaping. *Rationale and Process*: A key condition for a norm to exist is that a respondent believes that others know that there is a norm to perform some behavior and, critically, that others expect them to adhere to the norm. Here we elicit the beliefs of the respondent about that expectation (49). We use this to assess the impact of the intervention on the beliefs about those expectations and therefore on the norms about smoking. Since we are conducting experiments before and after the smoking prevention interventions, we wish to avoid, as far as possible, ascertaining shifts in attitudes that are consequences of merely “learning the right answer” (what the adolescents might believe adults want to hear) as opposed to tapping into the implicit belief norms. This is not a concern in the pre-intervention stage. The actions/behaviors rated in the game are the same actions for which we elicit descriptive norms.

##### Measuring willingness to pay to support anti-smoking norms

Just as a willingness to incur costs to follow the rule in the RF task reveals a respect for norms more generally, a willingness to incur a cost to encourage smoking reduction by others reveals support for anti-smoking norms. Thus, we asked participants to make a donation decision in which they were allocated a sum of money (tokens worth £5.00 in Northern Ireland and £2.30 in Colombia) and they must decide how much of that money they want to keep for themselves and how much they want to donate to the organizations responsible for ASSIST or Dead Cool. Participants were informed that the donations will help ensure that other pupils are more likely to be exposed to these anti-smoking interventions, and thus a donation reveals the participant's belief that such interventions are normatively appealing and effective, providing evidence for a behavioral impact of an injunctive anti-smoking social norm. Participants made the donation decision twice, once *before* the intervention and once *after* the intervention and were told that we would flip a coin to determine which of the two decisions is implemented. Participants' final payments (and donations) depended on the randomly chosen decision. Performing the task twice allowed us to determine if exposure to the intervention increases willingness to donate, and choosing to implement only one of the two decisions ensures that participants have incentives to report their willingness to donate truthfully at both decision times. We would additionally predict a larger effect among those who were more rule-following in the bucket task (i.e., among those who care more about following social norms).

#### Smoking Behavior

At both time-points, pupils had their smoking behavior in the last week measured using a hand-held carbon monoxide monitor (administered by a trained member of the research team). A hand-held PICOAdvantage Smokerlyzer (Bedfont) was used to measure expelled air carbon monoxide from the pupils at the two testing time points. This is an electrochemical sensor which measures carbon monoxide in parts per million (ppm). It measures a range of 0–150 ppm with an accuracy of 2 ppm/5% (whichever is greater).

#### Self-Report Outcomes

Pupils were asked to complete a survey before and after the smoking prevention interventions had taken place to assess their self-reported smoking behaviors and attitudes. Questionnaire items are summarized in Supplementary File 1. All questionnaire items have been validated and adopted from previous studies conducted with children of a similar age. Surveys were delivered via Qualtrics (www.Qualtrics.com) and collected using tablet computers in each school.

##### Socio-demographics

Demographic information was collected in the baseline survey. This included gender, age, home postcode (to capture individual level deprivation based on country specific area level deprivation measures) in Northern Ireland and address to assess socio-economic position from the household, ethnicity and detail on who the pupil lives with (i.e., mother, father, step-parent, sisters/brothers, foster care, other).

##### Smoking behavior (past and present)

At baseline and 1 week after the end of the intervention period, pupils were asked four questions about their current and past smoking behavior. Each question has a unique scaled response appropriate to the question being asked and has been used in previous studies (37, 50).

##### Hypothesized mediators

A range of psychosocial constructs were collected at baseline and 1 week after the end of the ASSIST/Dead Cool interventions. ***Smoking intentions/susceptibility*** were assessed with four questions asking the pupils: (1) if they **do** currently smoke, do they intend to quit smoking in the next 6 months (response on a five-point Likert scale from “Definitely remain a smoker” to “Definitely quit” with an additional response option for “I don't smoke”); (51) (2) do they think they will try a cigarette soon (response “Yes,” “No,” “Don't know”); (37, 52) (3) if one of their best friends were to offer them a cigarette would they smoke it (response on a five-point Likert scale from “Definitely yes” to “Definitely not”); (37, 52) (4) if they **don't** currently smoke, do they intend to take up smoking in the next 6 months (response on a five-point Likert scale from “Definitely remain a non-smoker” to “Definitely start smoking” with an additional response option for “I am a smoker”). (51) ***Self-efficacy*** was assessed using the Lawrance (53) adaptation of the scales outlined in Condiotte and Lichtenstein (54) (with three subscales: Emotional, Friends, Opportunity). ***Perceived risks of tobacco-use*** were assessed using the scales outlined in Halpern-Felsher et al. (55), Song et al. (56), and Aryal et al. (57) (with three subscales: Physical, Social, Addiction). ***Perceivedbenefits of tobacco-use*** were assessed using the scales outlined in Halpern-Felsher et al. (55), Song et al. (56), and Aryal et al. (57) (with two subscales: Physical, Social). ***Perceived behavioral control*** was assessed with two questions asking the pupils: (1) how easy they think it would be for them to quit smoking if they smoked regularly (i.e., difficulty to quit); (2) if they decided not to smoke, how sure are they that they could avoid smoking (i.e., avoid smoking) (responses on a five-point Likert scale from “Strongly disagree” to “Strongly agree”) (58). ***Attitudes towardsmoking*** was assessed with the 12-items scale outlined in Ganley and Rosario (59). ***Knowledge of smoking*** was assessed with the six-items scale outlined in Cremers et al. (60). ***Social Norms (injunctive norms)*** and ***Social modeling (descriptivenorms)*** were assessed with the scales outlined in Cremers et al. (60). ***Exposure toadvertising in the media*** was assessed with the 7-items scale outlined in Stigler et al. (61) in Northern Ireland and using a 12-item scale in Colombia, according to the advertising context. An additional item was used to assess ***Exposure to advertising inshops*** (37).

##### Social networks and pro-sociality

At baseline and 1 week after the end of the intervention period, pupils were asked questions about social networks in their year group (i.e., closest school friends (37), relationship quality, friends who he/she spends most time with outside of school, influential peers) (24). The following final question was added only at follow-up “Can you remember having any conversations with your friends about the risks and benefits of smoking over the past 6 months? If so, how many and with whom?” Pupils were provided with a school year roster in order to help with completion of the social network nominations. Pro-sociality will be assessed with the Need to Belong Scale (62), Fear of Negative Evaluation Scale (63, 64), and Pro-Social Behavior Scale (65).

##### Well-being, absenteeism, other

We assessed the adolescent personality traits (Openness, Extraversion, Agreeableness, Conscientiousness, Emotional Stability) using the *Big Five Trait Short Questionnaire* (BFPTSQ) (66). In Northern Ireland we applied the scaled validation by Morizot (66) and in Colombia we applied the scale validated by Orlet (67). Self-perceived well-being was assessed using the scale developed by the Children's Society, and based on Huebner's life satisfaction scale (37, 68). Rebelliousness and sensation seeking was assessed with the four questionnaire items described in Russo et al. (69); and Dunne et al. (37). Truancy, school education on smoking, and access to and disposal of pocket money was assessed using five questions adapted from Dunne et al. (37).

### Statistical Methods

#### Elicitation of Social Norms and Social Network Analyses

Injunctive social norms will be modeled quantitatively, such that a decision maker's “pay-off” u(a_k_) from a set of actions V {π(a_k_)} is related to the parameter γ ≥ 0, representing the degree to which the individual cares about adhering to social norms, in: u(a_k_) = V {π(a_k_)} + γN(a_k_), with the function N capturing the social norm, [i.e., the social appropriateness of action a_k_ denoted by N(a_k_)]. We let N_g_(a_k_) denote the social norms for group *g*, estimated from the Coordination games. γ is the key parameter reflecting individual sensitivity to the norm, estimated using the total number of balls allocated to the blue “rule following” bucket in the RF task. The Dictator and Coordination games allow us to identify norms related to prosocial behavior and to smoking related activities in the target population. We can then explore whether the norms related to smoking actually do change and whether data from the RF experiments help us predict who is more sensitive to following those changed norms after the intervention. We will also measure correlations (Spearman's rank correlation) between an individual's norms-sensitivity parameter and his/her decisions in the Donation game. We will use tobit regressions that allow for censoring, regressing the distance (Δ_SNdist_) between an individual's choice and the observed average norm (average Dictator choice) in the relevant reference group on his/her norm-sensitivity parameter. The tobit regression allows us to see if there is a statistically significant relationship between norm-sensitivity and the changed norms after the intervention. We will use similar techniques when examining social norms related to tobacco use and individuals' abilities to identify them in the Coordination game. For each behavioral vignette (whose appropriateness is rated), we will calculate a correlation between individuals' norms-sensitivity parameters, and their chosen score on the social appropriateness scale, after calculating (Δ_SNdist_). Scores on the appropriateness scale are categorical, requiring ordered probit regressions. We will conduct the above analysis both pre- and post-intervention but since we will have two observations for each individual, we will use the respective panel random effects versions (including a dummy variable to identify post-intervention observations). In all regressions, we will calculate standard errors clustered on school classes. We will account for individual participants' demographic and psychosocial characteristics, and dummy variables signifying intervention type (ASSIST or Dead Cool intervention) and country (Colombia or Northern Ireland).

${\text{H}}_{0}^{1}$**: that individual psychosocial traits and susceptibility to social norms are independent**: After checking distributional assumptions, multiple linear regression (and where appropriate analysis of variance; ANOVA) will be employed, with any necessary transformations of raw variables; a sample size of 600 in each country achieves 80% power to detect a correlation coefficient of 0.16 for the association between susceptibility to social norms and a continuous psychosocial trait, after adjusting for a design effect of 1.99, assuming a sample of 100 per school and intra-class correlation coefficient (ICC) = 0.01.

${\text{H}}_{0}^{2}$**: that the individual social norms susceptibilities among friendship cliques are uncorrelated**: the analytic approach in this case will be similar to that for ${\text{H}}_{0}^{5}$ which is illustrated in the section sample size for the purpose of power calculation.

${\text{H}}_{0}^{3}$**: that social norms at the class and year group level are independent of social network structure**: a novel aspect of this proposal is that we will examine how social norms sensitivities (Δ_SNdist_) cluster among friendship groups (before and after the two interventions), and thus examine possible mediating mechanisms for the intervention effects and whether they are moderated by social network structure. We will extend the clustering method presented by Grimmer and King (70), using a Markov Chain Monte Carlo (MCMC) model to integrate various attributes of the students and their social norms sensitivity parameters in the first step of the *a posteriori* clustering selection framework. Examining the influence of individual student attributes (their norms sensitivities; psychosocial traits like self-efficacy, and the number of family members who smoke) within a MCMC framework permits inference based on well-known expectation and maximization algorithms. We note that peer influence was inferred using hidden Markov models (7) applied to *before* and *after* data from three ASSIST schools, but with a similar per country sample size as Steglich et al. (7), we will obtain superior experimental measures of norms and thus populate the MCMC models with real rather than inferred parameters. If homophily is not stable over one semester, in a sensitivity analysis we will use an instrumental variable approach, instrumenting on orthogonal characteristics of students not nominated by the ego but who were friends of their friends, using characteristics that should not plausibly predict the egos' norms sensitivity (Δ_SNdist_), nor friendship structure (71).

${\text{H}}_{0}^{4}$**: that the changes in smoking related norms are the same in schools which were offered the ASSIST intervention and in those which were offered the Dead Cool intervention**: we will use a regression based approach permitting adjustment for the multilevel clustered nature of the data, comparing the change in smoking related norms (identified through the Coordination games) in ASSIST and Dead Cool Schools and between UK and Colombian Schools.

${\text{H}}_{0}^{5}$**: that any changes in social norms are independent among members of the same friendship clique**: see section sample size.

#### Mediation Analyses

The relationship between intervention (ASSIST vs. Dead Cool) and smoking related norms (${\text{H}}_{0}^{4}$) will be examined for mediation by the psychosocial traits (i.e., mediators) using the “Cross-lagged panel model for a half-longitudinal design” described by Preacher (72). In each model, a variable indicating intervention group (i.e., ASSIST vs. Dead Cool) will be modeled as a predictor of the mediator at time 2 (i.e., intervention-end); the mediator at time 1 (i.e., baseline) will be modeled as a predictor of the mediator at time 2 and smoking related norms at time 2; smoking related norms at time 1 will be modeled as a predictor of smoking related norms at time 2. Time 1 variables will be permitted to covary (72). The significance of indirect effects will be assessed using the structural equation modeling (SEM)-based product-of-coefficients approach (73) with 95% confidence intervals (CIs) estimated using the bias-corrected bootstrap (with 10,000 iterations) procedure (74, 75). Model fit will be assessed using recommended indices and cut-points (76). These analyses will be repeated to examine the relationship between mediators and smoking behavior/intentions.

#### Process Evaluation Analyses

In a final qualitative phase, we will evaluate the findings from our quantitative analysis of social norms, and their changes post intervention, by triangulating with data from focus groups with students (one focus group per school with a diverse sample of ~8 students each) and interviews with the designated peer leaders (in the schools receiving the ASSIST intervention) and with teachers (in schools receiving both interventions). Qualitative analysis will draw on the framework approach. Each intervention site will be treated as a “case” and within case analysis conducted by use of framework matrices. A *cross case* comparison will then be made by charting common themes and identifying key mechanisms of action (seeking comparisons across interventions and across countries). Our triangulation approach will first involve sorting of themes; convergence coding (for agreement/partial agreement, silence and dissonance); feedback and interpretative synthesis (77). The overall interpretation of this mixed methods triangulation (and the “proof of concept”) will be tested in a plenary workshop of stakeholders (from public health, education, network science and behavioral economics) using Group Model Building, a participatory approach that is widely used to build the capacity of practitioners to think in a systems way and which employs nominal group techniques to uncover hidden assumptions and build consensus around the possible mechanisms within a complex system (78).

## Discussion

This *proof of concept* study aims to fill a gap in the public health science literature. The research proposed is innovative because it harnesses novel transdisciplinary insights (from Game Theory and Social Network science) about the elicitation and measurement of social norms in order to better understand the effects of school based smoking prevention programs. However, the underpinning methodology will have a wider relevance for the study of other health-related behaviors and for understanding how social norms are mediated by social networks in classrooms and schools. It will provide insights into the psycho-social, cultural, and economic drivers of observed differences in smoking rates among adolescents in high and LMIC settings, producing new knowledge that can help reduce smoking in these settings. It is also significant in aspiring to a deeper understanding of possible mechanisms (through social norms) of *spill-over effects*. Finally, the research is important as it will help build transdisciplinary capacity in public health science in a LMIC setting, with clear pathways to impact. By invoking new approaches from Game Theory and Social Network science, the project will leave a legacy of transdisciplinary skills development in both LMIC and UK settings.

## Ethics Statement

The studies involving human participants were reviewed and approved. Ethical approval has been granted from the Queen's University Belfast, School of Medicine, Dentistry and Biomedical Sciences ethics committee (reference number 18.43; v3 Sept 2018), and Research committee of the Universidad de Los Andes, Bogotá (937 - July 30, 2018). Written informed consent to participate in this study was provided by the participants' legal guardian/next of kin.

## Author Contributions

FK, RH, FM, and OS had the idea for the study and led the funding application. FK is responsible for overall project oversight, line management of research staff, dissemination activities, administration of funds, and submitting reports. OS undertakes a similar role in Colombia. RH assumes the role of de facto joint PI, and supports FK in terms of staff and day-to-day management of the project, as well as providing specific expertise in network science and complex interventions. FM assumes a similar role to RH in supporting OS, and providing specific expertise in social network analysis. RK will be responsible for the development and pilot testing of the protocols for the game theory experiments, and will be assisted by AR, EKr, and EKi. This team of behavioral economic experts will also lead and advise on the analysis of these experiments. LD will lead the development of the qualitative and process evaluation protocols and assist with the analyses of this data. HZ will assist RH and FM with the social network analyses. LM and LB will assist with quality assurance, intervention fidelity, and provide important links between research, practice, and policy. One Postdoctoral Researcher will be employed at QUB (JM) and a Psychologist with Masters on public Health from UniAndes (SS-F). They are responsible for the day-to-day management of the project, supervised by the PIs. They are involved in all aspects of development of the study materials, piloting the study protocols, overseeing recruitment, data collection, data entry, and cleaning, and will be involved in the data analyses, taking advice from the specific team experts in game theory, and network science. Two Research Assistants at QUB (SM) and UniAndes (JJ) will assist with recruitment and data collection, and will undertake the qualitative research element. All authors contributed to the article and approved the submitted version.

## Conflict of Interest

The authors declare that the research was conducted in the absence of any commercial or financial relationships that could be construed as a potential conflict of interest.
