# Supplementary material for: MECHANISMS Study: Using Game Theory to Assess the Effects of Social Norms and Social Networks on Adolescent Smoking in Schools—Study Protocol
Source: Front Public Health. 2020 Aug 4;8:377. doi: 10.3389/fpubh.2020.00377 (PMC7417659; doi:10.3389/fpubh.2020.00377)
Supplement: Supplementary file 1 [file Table_1.DOCX]

**Supplementary File 1. Questionnaire items**

| Concept | Items (n) | Example question item and example answering options | Time-points | Reference(s) |
| --- | --- | --- | --- | --- |
| ***Socio-Demographics*** | | | | |
| Sex | 1 | Are you a boy or a girl? *Boy* (1), *Girl* (2). | Baseline | Fuller & Hawkins, 2012;^1^ Dunne et al., 2016^2^ |
| Age | 1 | How old are you now? *11 years old* (1) to *14 years old* (4).  In Bogota: plus *15 or more years old* (5)*.* | Baseline | Fuller & Hawkins, 2012;^1^ Dunne et al., 2016^2^ |
| Address/Postcode | 2 | What is your address and your postcode e.g. BT12 8NF?  In Bogotá: What is your address (for example: Cr 1 # 3-20) | Baseline | Dunne et al., 2016^2^ |
| Ethnicity | 1 | What is your ethnic group? Tick one box only, the one that is most true for you. E.g. *White British* (1), *African* (2).  In Bogota: According to your culture, town or physical features, you are or your recognize yourself as: E.g. I*ndigenous* (1)*, Gypsy/Rom* (2)*, None of them* (6) | Baseline | Fuller & Hawkins, 2012;^1^ Dunne et al., 2016^2^  DANE (2019)^3^ |
| Family | 1 | Who do you live with? (please tick all that apply). E.g. *Mother* (1) *Sisters/brothers* (2). | Baseline | Dunne et al., 2016^2^ |
| ***Smoking Behavior (Past and Present)*** | | | | |
| Current smoking behavior | 1 | Do you smoke cigarettes at all nowadays? *Yes* (1), *No* (2). | Baseline, intervention-end | Fuller & Hawkins, 2012;^1^ Dunne et al., 2016^2^ |
| Past/current smoking behavior | 1 | Now read the following statements carefully and tick the box next to the one which best describes you. *I have never smoked* (1), *I have only ever tried smoking once* (2). *I used to smoke sometimes but I never smoke a cigarette now* (3)*, I sometimes smoke cigarettes now but I don’t smoke as many as one a week* (4) | Baseline, intervention-end | Fuller & Hawkins, 2012;^1^ Dunne et al., 2016^2^ |
| Past/current smoking behavior | 1 | Just to check, read the statements below carefully and tick the box next to the one which best describes you. *I have never tried smoking a cigarette, not even a puff or two* (1), *I did once have a puff or two of a cigarette, but I never smoke now* (2). *I do sometimes smoke cigarettes* (3) | Baseline, intervention-end | Fuller & Hawkins, 2012;^1^ Dunne et al., 2016^2^ |
| Current smoking behavior | 1 | If you DO smoke, please indicate how many cigarettes you smoke. *I usually smoke between one and six cigarettes a week* (1), *I usually smoke more than six cigarettes a week* (2), *I don’t smoke* (3), *How many cigarettes do you smoke in a week?……* (4). | Baseline, intervention-end | Fuller & Hawkins, 2012;^1^ Dunne et al., 2016^2^ |
| Age at first smoking | 1 | If you DO smoke, how old were you when you first tried smoking a cigarette, even if it was only a puff or two? Write your age in the box. *I was …… years old* (1), *I don’t smoke* (2)*.* | Baseline, intervention-end | Fuller & Hawkins, 2012;^1^ Dunne et al., 2016^2^ |
| ***Mediators*** | | | | |
| Smoking intentions/Susceptibility | 1 | This question is about your intentions towards smoking. If you **DO** currently smoke, do you intend to quit smoking in the next six months? *Definitely remain a smoker* (1) to *I don’t smoke* (6). | Baseline, intervention-end | Mazanov et al., 2007^4^ |
| Smoking intentions/Susceptibility | 1 | This question is about your intentions towards smoking. Do you think you will try a cigarette soon? *Yes* (1), *No* (2), *Don’t know* (3). | Baseline, intervention-end | Pierce et al., 1998;^4^ Dunne et al., 2016^2^ |
| Smoking intentions/ Susceptibility | 1 | This question is about your intentions towards smoking. If one of your best friends were to offer you a cigarette, would you smoke it? (Please tick your answer). *Definitely yes* (1) to *Not sure* (5). | Baseline, intervention-end | Pierce et al., 1998;^5^ Dunne et al., 2016^2^ |
| Smoking intentions/ Susceptibility | 1 | This question is about your intentions towards smoking. If you **DON’T** currently smoke, do you intend to take up smoking in the next 6 months? (Please tick your answer). *Definitely remain a non-smoker* (1) to *I am a smoker* (6). | Baseline, intervention-end | Mazanov et al., 2007^4^ |
| Self-efficacy (Emotional) | 9 | How sure are you that you could resist smoking cigarettes when you are uptight? *I am very sure I would smoke* (1) to *I am very sure I would NOT smoke* (6). | Baseline, intervention-end | Condiotte & Lichtenstein, 1981;^6^ Lawrance, 1989^6^ |
| Self-efficacy (Friends) | 9 | How sure are you that you could resist smoking cigarettes when you are at a friend's house, no adults are home? *I am very sure I would smoke* (1) to *I am very sure I would NOT smoke* (6). | Baseline, intervention-end | Condiotte & Lichtenstein, 1981;^5^ Lawrance, 1989^7^ |
| Self-efficacy (Opportunity) | 11 | How sure are you that you could resist smoking cigarettes when you are playing video games? *I am very sure I would smoke* (1) to *I am very sure I would NOT smoke* (6). | Baseline, intervention-end | Condiotte & Lichtenstein, 1981;^6^ Lawrance, 1989^7^ |
| Perceived risks of tobacco-use (Physical) | 7 | Imagine that you just began smoking. You smoke about 2 or 3 cigarettes each day. Sometimes you smoke alone, and sometimes you smoke with friends.  Please estimate the chance you will get the following risks by using any percentage between 0% and 100% (where 0%='No chance', 100%='Certain')? You will get lung cancer? *0%* (No chance) to *100%* (Certain). | Baseline, intervention-end | Halpern-Felsher et al., 2004;^8^ Song et al., 2009;^9^ Aryal et al. 2013^10^ |
| Perceived risks of tobacco-use (Social) | 3 | Imagine that you just began smoking. You smoke about 2 or 3 cigarettes each day. Sometimes you smoke alone, and sometimes you smoke with friends.  Please estimate the chance you will get the following risks by using any percentage between 0% and 100% (where 0%='No chance', 100%='Certain')? You will get into trouble? *0%* (No chance) to *100%* (Certain). | Baseline, intervention-end | Halpern-Felsher et al., 2004;^8^ Song et al., 2009;^9^ Aryal et al. 2013^10^ |
| Perceived risks of tobacco-use (Addiction) | 3 | Imagine that you just began smoking. You smoke about 2 or 3 cigarettes each day. Sometimes you smoke alone, and sometimes you smoke with friends.  Please estimate the chance you will get the following risks by using any percentage between 0% and 100% (where 0%='No chance', 100%='Certain')? You will become addicted to cigarettes? *0%* (No chance) to *100%* (Certain). | Baseline, intervention-end | Halpern-Felsher et al., 2004;^8^ Song et al., 2009;^9^ Aryal et al. 2013^10^ |
| Perceived benefits of tobacco-use (Physical) | 3 | Imagine that you just began smoking. You smoke about 2 or 3 cigarettes each day. Sometimes you smoke alone, and sometimes you smoke with friends.  Please estimate the chance you will get the following benefits by using any percentage between 0% and 100% (where 0%='No chance', 100%='Certain')? You will feel relaxed after smoking? *0%* (No chance) to *100%* (Certain). | Baseline, intervention-end | Halpern-Felsher et al., 2004;^8^ Song et al., 2009;^9^ Aryal et al. 2013^10^ |
| Perceived benefits of tobacco-use (Social) | 2 | Imagine that you just began smoking. You smoke about 2 or 3 cigarettes each day. Sometimes you smoke alone, and sometimes you smoke with friends.  Please estimate the chance you will get the following benefits by using any percentage between 0% and 100% (where 0%='No chance', 100%='Certain')? You will look cool? *0%* (No chance) to *100%* (Certain). | Baseline, intervention-end | Halpern-Felsher et al., 2004;^8^ Song et al., 2009;^9^ Aryal et al. 2013^10^ |
| Perceived behavioral control (difficulty to quit) | 1 | Please indicate the extent to which you agree or disagree with the following statements. If I smoked regularly, I'm sure that it would be easy for me to quit. *Strongly disagree* (1) to *Strongly agree* (5). | Baseline, intervention-end | Smith et al., 2006^11^ |
| Perceived behavioral control (avoid smoking) | 1 | Please indicate the extent to which you agree or disagree with the following statements. If I decided not to smoke, I am sure I could avoid smoking. *Strongly disagree* (1) to *Strongly agree* (5). | Baseline, intervention-end | Smith et al., 2006^11^ |
| Attitudes towards smoking | 12 | This question is about your attitudes towards smoking. Please indicate the extent to which you agree or disagree with the following statements. Smoking looks cool. *Strongly disagree* (1) to *Strongly agree* (5). | Baseline, intervention-end | Ganley & Rosario 2013^12^ |
| Knowledge of smoking | 6 | This question is about your knowledge of smoking. Please indicate whether you agree or disagree with the following statements. Smoking causes your skin to age faster. *Yes* (1), *No* (2), *I don’t know* (3). | Baseline, intervention-end | Cremers et al., 2012^13^ |
| Social norms (injunctive norms) | 7 | Please indicate whether you think the following people would approve or disapprove if you were to start smoking. Most of the people who are important to me think that I… *Definitely should not smoke* (1) to *Definitely should smoke* (5). | Baseline, intervention-end | Cremers et al., 2012^13^ |
| Social modelling (descriptive norms) | 8 | Please indicate whether you are aware that the following people smoke. Does your best friend smoke? *Very often* (1) to *I don’t have a best friend* (7). | Baseline, intervention-end | Cremers et al., 2012^13^ |
| Exposure to advertising (media) | 8 | Have you seen any advertisements for tobacco in movies? *No* (1), *Yes* (2). | Baseline, intervention-end | Stigler et al., 2006^14^ with an additional response category for "computer games" |
| Exposure to advertising (shops) | 1 | In the past year, have you seen cigarette packets on display in any of the shops listed below? Please select all that apply. *A supermarket* (1), *A newsagent, tobacconist or a sweet shop* (2). | Baseline, intervention-end | Dunne et al., 2016^2^ |
| ***Social Networks/Pro-Sociality*** | | | | |
| Closest school friends | 1 | For these questions, please print the full name of any pupil you want to suggest. You will be provided with a school year roster to help you. Each pupil must be in your year group at your school. Don't name more than ten pupils. Can you name up to ten your closest friends in your school year? | Baseline, intervention-end | Dunne et al., 2016^2^ |
| Relationship quality | 1 | For these questions, please print the full name of any pupil you want to suggest. You will be provided with a school year roster to help you. Each pupil must be in your year group at your school. Don't name more than ten pupils. Among your school peers, with whom would you talk to about something upsetting to you? | Baseline, intervention-end | N/A |
| Friends who you spend time with outside of school | 1 | Can you name up to ten friends from your school year who you spend time with outside of school? | Baseline, intervention-end | N/A |
| Influential peers | 2 | For this question, please print the full name of any pupil you want to suggest. You will be provided with a school year roster to help you. Each pupil must be in your year group at your school. Who do you respect in your year at your school? Only list one pupil from your school year.  Who do you identify as influential in your school? Only list one pupil from your school year. | Baseline, intervention-end | Starkey et al., 2005^15^ |
| Need to Belong Scale | 10 | Please indicate the degree to which each of the following statements is true or characteristic of you. If other people don’t seem to accept me, I don’t let it bother me. *Not at all* (1) to *Extremely* (5). | Baseline, intervention-end | Leary et al., 2013;^16^ Bevelander et al., 2018^17^ |
| Fear of Negative Evaluation Scale | 12 | Read each of the following statements carefully and indicate how characteristic it is of you according to the following scale. I worry about what other people will think of me even when I know it doesn't make any difference. *Not at all characteristic of me* (1) to *Extremely characteristic of me* (5). | Baseline, intervention-end | Leary, 1983;^18^ Collins et al., 2005;^19^ Bevelander et al., 2018^17^ |
| Pro-social behavior | 5 | For each item, please mark the box for Not True, Somewhat True or Certainly True. Please give your answers on the basis of how things have been for you over the last six months. I try to be nice to other people. I care about their feelings. *Not true* (1), *Somewhat true* (2), *Certainly true* (3). | Baseline, intervention-end | Goodman et al., 2003;^20^ Bevelander et al., 2018^17^ |
| ***Wellbeing, Absenteeism, Other*** | | | | |
| “Big Five” Personality Traits (Five subscales: Openness, Extraversion, Agreeableness, Conscientiousness, Emotional Stability) | 50 | Please say how much you agree or disagree with the following sentences. I see myself as someone who is original, often has new ideas. *Totally disagree* (0) to *Totally agree* (4). | Baseline, intervention-end | Morizot, 2003^21^  In Bogota: Ortet et al., 2017^22^ |
| Wellbeing | 5 | Please say how much you agree or disagree with the following sentences (Please tick your answer). My life is going well. *Strongly agree* (1) to *Don’t know* (6). | Baseline, intervention-end | Rees et al., 2010;^23^ Dunne et al., 2016^2^ |
| Rebelliousness and sensation seeking | 4 | How much do the following statements describe you? (Please tick your answer). I get in trouble in school. *Exactly like me* (1) to *Not at all like me* (4). | Baseline, intervention-end | Russo et al., 1993;^24^ Dunne et al., 2016^2^ |
| Truancy | 1 | Have you ever stayed away from school without permission (truanted/bunked off)? *Yes* (1), *No* (2), *Don’t know* (3). | Baseline, intervention-end | Dunne et al., 2016^2^ |
| School education on smoking | 2 | Do you think school has given you enough information on smoking? *Yes* (1), *No* (2), *Don’t know* (3). | Baseline, intervention-end | Dunne et al., 2016^2^ |
| Intervention awareness | 1 | Can you remember having any conversations with your friends about the risks and benefits of smoking over the past six months? If so, how many?  Please name up to 10 people in your school year that you talked to or who talked to you about smoking and/or vaping since the start of our project. | Intervention-end  Intervention-end | N/A  N/A |
| Access to and disposal of pocket money | 2 | Do you get regular pocket money (from parents, relatives?). *Yes* (1), *No* (2). | Baseline, intervention-end | Cremers et al., 2012;^13^ Dunne et al., 2016^2^ |

**References (supplementary file)**

^1^ Fuller E, Hawkins V. *Smoking, drinking and drug use among young people in England in 2011*. London: Health and Social Care Information Centre; 2012

^2^ Dunne L, Thurston A, Gildea A, Kee F, Lazenbatt A. Protocol: A randomised controlled trial evaluation of Cancer Focus NI’s ‘Dead Cool’ smoking prevention programme in post-primary schools. *Int J Educ Res*. 2016;75:24-30.

^3^ Departamento Administrativo Nacional de Estadísticas (DANE) (2019). Información de los grupos étnicos en Colombia.. Bogotá, Colombia <https://www.dane.gov.co/index.php/estadisticas-por-tema/demografia-y-poblacion/grupos-etnicos/estadisticas-y-grupos-etnicos/definicion-de-preguntas>

^4^ Mazanov J, Byrne DG. “‘Do you intend to smoke?’”: A test of the assumed psychological equivalence in adolescent smoker and nonsmoker intention to change smoking behaviour. *Aust J Psychol*. 2007;59(1):34-42.

^5^ Pierce JP, Choi WS, Gilpin EA, Farkas AJ, Berry CC. Tobacco industry promotion of cigarettes and adolescent smoking. *JAMA*. 1998;279(7):511-515.

^6^ Condiotte MM, Lichtenstein E. Self-efficacy and relapse in smoking cessation programs. *J Consult Clin Psychol*. 1981;49(5):648-658.

^7^ Lawrance L. Validation of a self-efficacy scale to predict adolescent smoking. *Heal Educ Res Theory Pract*. 1989;4(3):351-360.

^8^ Halpern-Felsher BL, Biehl M, Kropp RY, Rubinstein ML. Perceived risks and benefits of smoking: differences among adolescents with different smoking experiences and intentions. *Prev Med*. 2004;39(3):559-567.

^9^ Song A V., Morrell HER, Cornell JL, Ramos ME, Biehl M, Kropp RY, Halpern-Felsher BL. Perceptions of smoking-related risks and benefits as predictors of adolescent smoking initiation. *Am J Public Health*. 2009;99(3):487-492.

^10^ Aryal UR, Petzold M, Krettek A. Perceived risks and benefits of cigarette smoking among Nepalese adolescents: a population-based cross-sectional study. *BMC Public Health*. 2013;13:187.

^11^ Smith BN, Bean MK, Mitchell KS, Speizer IS, Fries EA. Psychosocial factors associated with non-smoking adolescents’ intentions to smoke. *Health Educ Res*. 2006;22(2):238-247.

^12^ Ganley BJ, Rosario DI. The smoking attitudes, knowledge, intent, and behaviors of adolescents and young adults: implications for nursing practice. *J Nurs Educ Pract*. 2013;3(1):40.

^13^ Cremers HP, Mercken L, Oenema A, de Vries H. A web-based computer-tailored smoking prevention programme for primary school children: intervention design and study protocol. *BMC Public Health*. 2012;12(1):277.

^14^ Stigler MH, Perry CL, Arora M, Reddy KS. Why are urban Indian 6th graders using more tobacco than 8th graders? Findings from Project MYTRI. *Tob Control*. 2006;15(Suppl 1):i54-60.

^15^ Starkey F, Moore L, Campbell R, Sidaway M, Bloor M. Rationale, design and conduct of a comprehensive evaluation of a school-based peer-led anti-smoking intervention in the UK: the ASSIST cluster randomised trial. *BMC Public Health*. 2005;5(1):43.

^16^ Leary MR, Kelly KM, Cottrell CA, Schreindorfer LS. Construct validity of the Need to Belong Scale: mapping the nomological network. *J Pers Assess*. 2013;95(6):610-624.

^17^ Bevelander KE, Smit CR, van Woudenberg TJ, Buijs L, Burk WJ, Buijzen M. Youth’s social network structures and peer influences: study protocol MyMovez project - Phase I. *BMC Public Health*. 2018;18(1):504.

^18^ Leary MR. A brief version of the Fear of Negative Evaluation Scale. *Personal Soc Psychol Bull*. 1983;9(3):371-375.

^19^ Collins KA, Westra HA, Dozois DJA, Stewart SH. The validity of the brief version of the Fear of Negative Evaluation Scale. *J Anxiety Disord*. 2005;19(3):345-359.

^20^ Goodman R, Meltzer H, Bailey V. The Strengths and Difficulties Questionnaire: a pilot study on the validity of the self-report version. *Int Rev psychiatry*. 2003;15(1-2):173-177.

^21^ Morizot J. Construct validity of adolescents’ self-reported Big Five personality traits. *Assessment*. 2014;21(5):580-606.

^22^ Ortet G, Martínez T, Mezquita L, Morizot J, Ibáñez M.I. Big Five Personality Trait Short Questionnaire: Preliminary validation with Spanish adults. *Spanish Journal of Psychology,* 2017; 20: 1–11.

^23^ Rees G, Bradshaw J, Goswami H, Keung A. *Understanding children’s well-being: A national survey of young people’s well-being*. London: Children’s Society; 2010.

^24^ Russo MF, Stokes GS, Lahey BB, et al. A sensation seeking scale for children: further refinement and psychometric development. *J Psychopathol Behav Assess*. 1993;15(2):69-86.
